# Measuring dynamic social contacts in a rehabilitation hospital: effect of wards, patient and staff characteristics

**DOI:** 10.1038/s41598-018-20008-w

**Published:** 2018-01-26

**Authors:** Audrey Duval, Thomas Obadia, Lucie Martinet, Pierre-Yves Boëlle, Eric Fleury, Didier Guillemot, Lulla Opatowski, Laura Temime, I-Bird study group, Anne Sophie Alvarez, Audrey Baraffe, Mariano Beiró, Inga Bertucci, Camille Cyncynatus, Florence Dannet, Marie Laure Delaby, Pierre Denys, Matthieu Domenech de Cellès, Antoine Fraboulet, Jean-Louis Gaillard, Jean-Louis Herrmann, Boris Labrador, Jennifer Lasley, Christine Lawrence, Judith Legrand, Odile Le Minor, Caroline Ligier, Karine Mignon, Catherine Sacleux, Jérôme Salomon, Marie Perard, Laure Petit, Laeticia Remy, Anne Thiebaut, Damien Thomas, Philippe Tronchet, Isabelle Villain

**Affiliations:** 1 Biostatistics, Biomathematics, Pharmacoepidemiology and Infectious Diseases (B2PHI), Inserm, UVSQ, Institut Pasteur, Université Paris-Saclay, Paris, France; 2 Institut Pasteur – Bioinformatics and Biostatistics Hub – C3BI, USR 3756 IP CNRS, Paris, France; 3 Malaria Parasites & Hosts Unit, Department of Parasites & Insect Vectors, Institut Pasteur, Paris, France; 4 ENS de Lyon, DANTE/INRIA, LIP UMR CNRS 5668 Université de Lyon, Lyon, France; 5 Sorbonne Universités, UPMC Univ Paris 06, UMR_S 1136, Institut Pierre Louis d’Epidémiologie et de Santé Publique, Paris, France; 6 ENS de Lyon, Université de Lyon, Laboratoire de l’Informatique du Parallélisme (UMR CNRS 5668- ENS de Lyon-UCB Lyon 1), IXXI Rhône Alpes Complex Systems Institute, Lyon, France; 7 INSERM 1181 Biostatistics, Biomathematics, Pharmacoepidemiology and Infectious Diseases (B2PHI), Institut Pasteur, B2PHI Paris, France; 8 Université de Versailles Saint-Quentin, UMR 1181, B2PHI, Montigny-Le-Bretonneux, France; 9 AP-HP, Raymond-Poincaré Hospital, Garche, France; 10 Laboratoire MESuRS, Conservatoire national des Arts et Métiers, Paris, France; 11 Unité PACRI, Institut Pasteur, Conservatoire national des Arts et Métiers, Paris, France; 12 AP-HP, Paris, France; 13 Universidad de Buenos Aires, Buenos Aires, Argentina; 14 AbAg, Chilly-Mazarin, France; 15 Insa, Lyon, France; 16 Inserm, Paris, France; 17 Univ Paris-Sud, UMR 0320/UMR8120 Génétique Quantitative et Evolution Le Moulon, Université Paris-Saclay, F-91190 Gif-sur-Yvette, France; 18 Institut Pasteur, Paris, France

## Abstract

Understanding transmission routes of hospital-acquired infections (HAI) is key to improve their control. In this context, describing and analyzing dynamic inter-individual contact patterns in hospitals is essential. In this study, we used wearable sensors to detect Close Proximity Interactions (CPIs) among patients and hospital staff in a 200-bed long-term care facility over 4 months. First, the dynamic CPI data was described in terms of contact frequency and duration per individual status or activity and per ward. Second, we investigated the individual factors associated with high contact frequency or duration using generalized linear mixed-effect models to account for inter-ward heterogeneity. Hospital porters and physicians had the highest daily number of distinct contacts, making them more likely to disseminate HAI among individuals. Conversely, contact duration was highest between patients, with potential implications in terms of HAI acquisition risk. Contact patterns differed among hospital wards, reflecting varying care patterns depending on reason for hospitalization, with more frequent contacts in neurologic wards and fewer, longer contacts in geriatric wards. This study is the first to report proximity-sensing data informing on inter-individual contacts in long-term care settings. Our results should help better understand HAI spread, parameterize future mathematical models, and propose efficient control strategies.

## Introduction

Each year, hundreds of millions of patients worldwide are affected by healthcare-associated infections (HAI), resulting in increased morbidity, mortality and costs^1^. This makes controlling the spread of HAI in hospitals a major public health issue. In order to control this spread, a better understanding of the routes of pathogen transmission within hospitals is required. This entails in particular a detailed description of the characteristics of inter-individual contact networks, in order to identify potential super-spreaders among healthcare workers and to propose preventive strategies and control measures against epidemic spread through the use of mathematical modelling approaches^2^.

Over the last decade, several studies have described contact patterns within human populations, most of which were based on self-reported approaches such as activity diaries^3,4^. However, this data has been shown to be biased^5^; one of the main issues being the lack of resolution, leading notably to under-reporting of short contacts which are very frequent in the hospital context. More recently, electronic wireless devices started being used to record close-proximity interactions (CPIs), notably in schools or hospitals^5–17^, providing accurate and detailed data on social interactions in these settings. To this date, hospital CPIs have mostly been recorded at the ward level, over short time periods; in order to fully understand the risk of HAI spread at the hospital level, the impact of different ward characteristics on contact networks, as well as inter-individual interactions across wards also need to be taken into account. In addition, most of previously published hospital CPI data was collected in acute-care settings. However, long-term care facilities (LTCF) have been shown to play a major part in the global spread of HAI, due notably to long patient lengths of stay^18,19^.

Here, we report data collected using Radio-Frequency Identification Devices (RFID) as wearable sensors to measure proximities with a high spatio-temporal resolution in a 200-bed LTCF, over a 4-month period. The objectives of this study are twofold. First, to provide a dynamic description of inter-individual contacts in different individual categories (patients and healthcare workers) and wards in the LTCF setting. Second, to identify factors associated with high contact levels in either patients or staff in order to inform future control programs in LTCF settings.

## Results

### Description of the population and study setting

**Figure 1:**
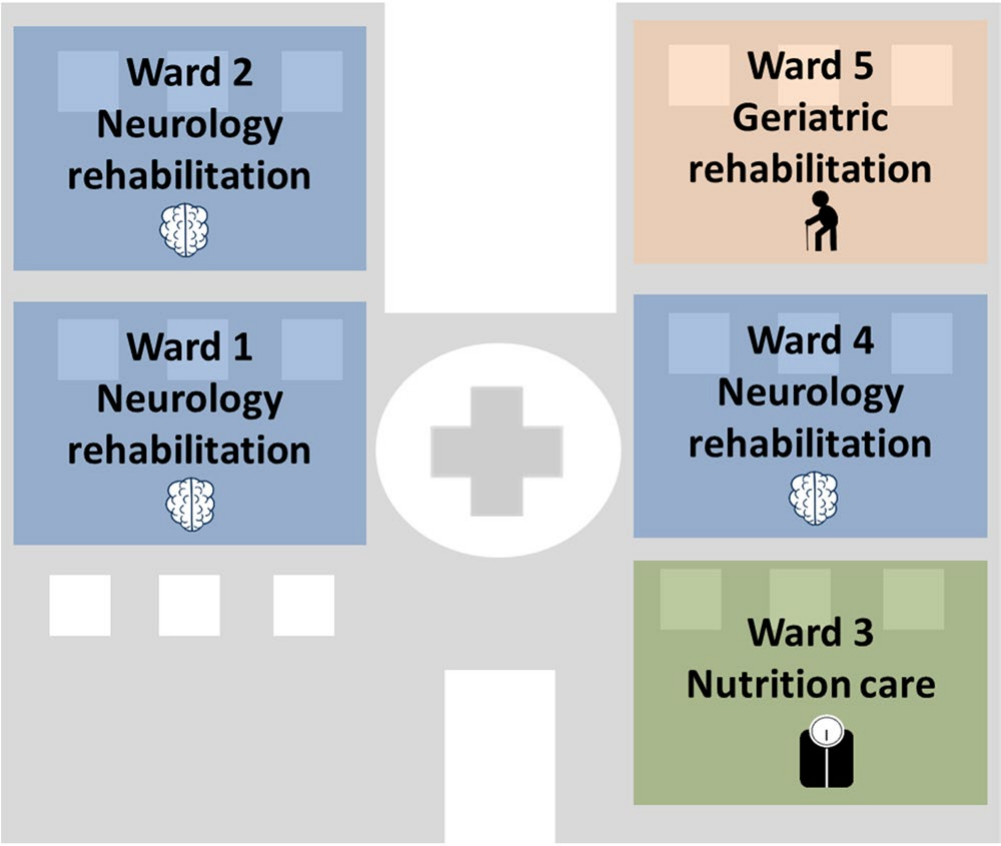
Organization of the Berck-sur-Mer hospital. The hospital is composed of five wards: 3 wards specialized in neurologic rehabilitation, 1 ward in geriatric rehabilitation and 1 ward in nutrition care.

I-Bird (Individual-Based Investigation of Resistance Dissemination) is a longitudinal study conducted in a 200-bed long-term and rehabilitation hospital in Berck-sur-Mer, France. The hospital is subdivided in 5 units corresponding to medical specialties (neurologic rehabilitation, obesity care and geriatric rehabilitation), mentioned as ward 1 (W1), ward 2 (W2), ward 3 (W3), ward 4 (W4) and ward 5 (W5) (Fig. 1). The study was conducted between May 1 and November 1, 2009, with the first two months serving as a pilot phase. The analyses presented here are restricted to the last 4 months of the study (July-October). On average, 136 patients and 174 hospital staff were present weekly over this time period. Hospital specificities included a long patient stay duration (seven weeks on average) and patient activities such as hair salon, cultural and artistic activities, reeducation care and balneotherapy with sea water.

In this paper, hospital staff was categorized in six groups: healthcare workers (HCW), including nurses, auxiliary nurses, nurse managers and nurse interns; reeducation staff, including physiotherapists and occupational therapists, ancillary hospital staff (AHS), physicians, hospital porters and logistic staff. Most of the staff was attached to one of the 5 wards; for some analyses, staff groups not administratively attached to a specific ward were grouped into the “transversal staff” category, in which hospital porter, logistic staff, as well as some mobile reeducation staff and HCWs, were brought together.

Overall, 2,671,832 CPIs were recorded and used for analysis. Among these CPIs, 2,279,515 (85.32%) involved patients, 944,142 (35.34%) HCWs, 94,100 (3.52%) reeducation staff, 109,789 (4.11%) AHS, 36,791 (1.38%) hospital porters and 33,406 (1.25%) physicians. Only 4,774 CPIs (0.18%) (respectively: 1,106 (0.04%)) involved animation staff (respectively: administrative staff).

### Distinct CPI frequency and duration by wards and categories

For a given individual in the hospital, the median (range) of the number of distinct daily CPIs contacts was 11.6 (1.6–47.3), and the median (range) of the daily cumulative duration of CPIs was 17.1 min (1.1–174.1).

**Figure 2:**
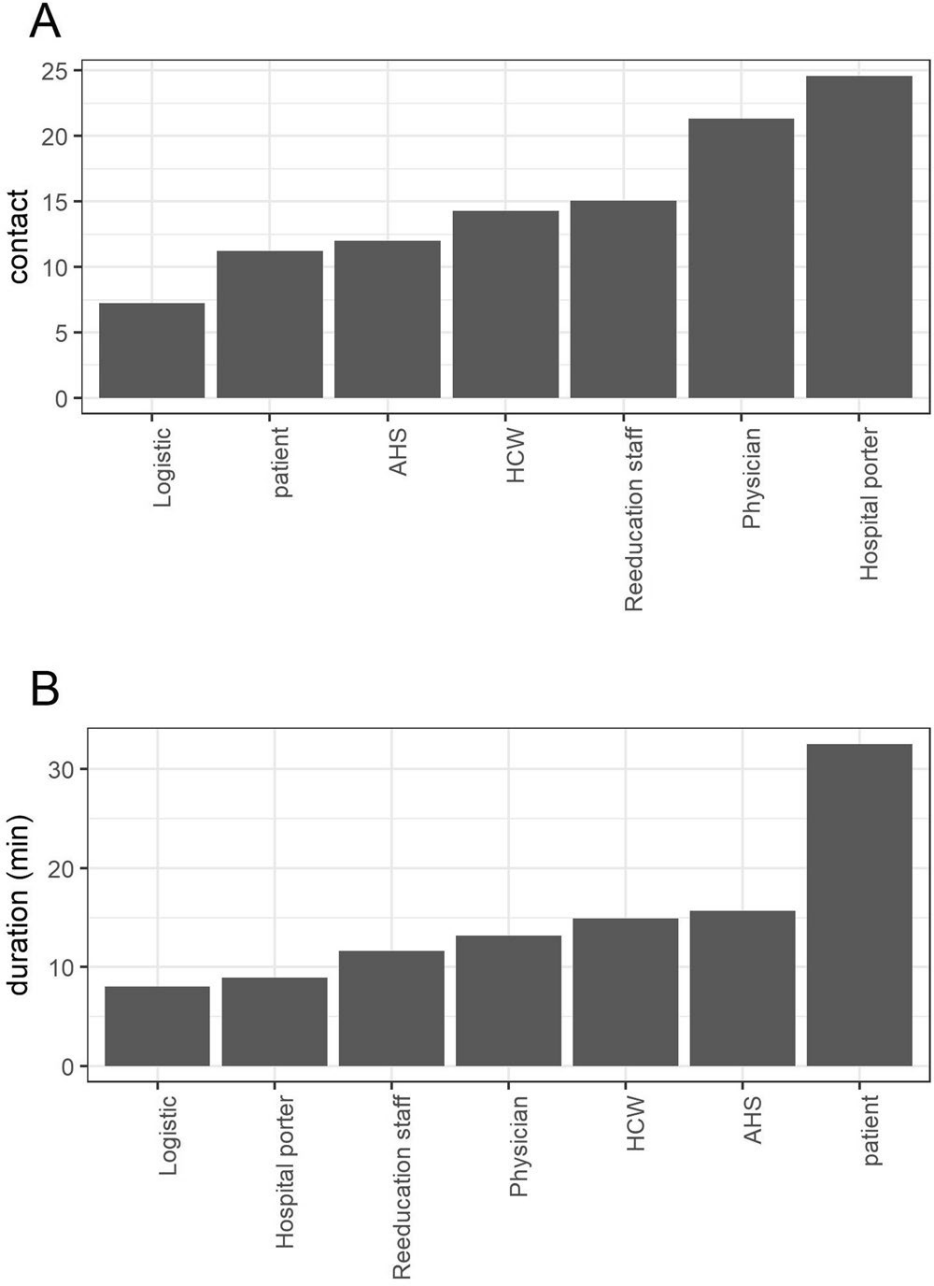
Number of (**A**) daily distinct CPIs and (**B**) daily cumulative duration of CPIs, per category. Here, daily distinct CPIs represent, for individuals of each category, the average number of distinct individuals met over a day (Supplementary Text S1). Daily cumulative duration of CPIs represents, for each category, the average total duration two individuals spend in contact with each other over one day (Supplementary Text S1).

The average number of daily distinct CPIs and the average daily cumulative duration of CPIs are reported in Fig. 2 for each category. CPIs were more frequent for hospital porters (24.6 distinct CPIs/day, 95% CI: 10.5–38.6), physicians (21.3 distinct CPIs/day, 95% CI: 13.5–28.6) and HCWs (14.3 distinct CPIs/day, 95% CI: 13.5–15) than for patients (11.2 distinct CPIs/day, 95% CI: 10.5–11.8) (Fig. 2A). However, the cumulative duration spent in contact was largest in patients (32 minutes/day, 95% CI: 29.7–35.3).

**Figure 3:**
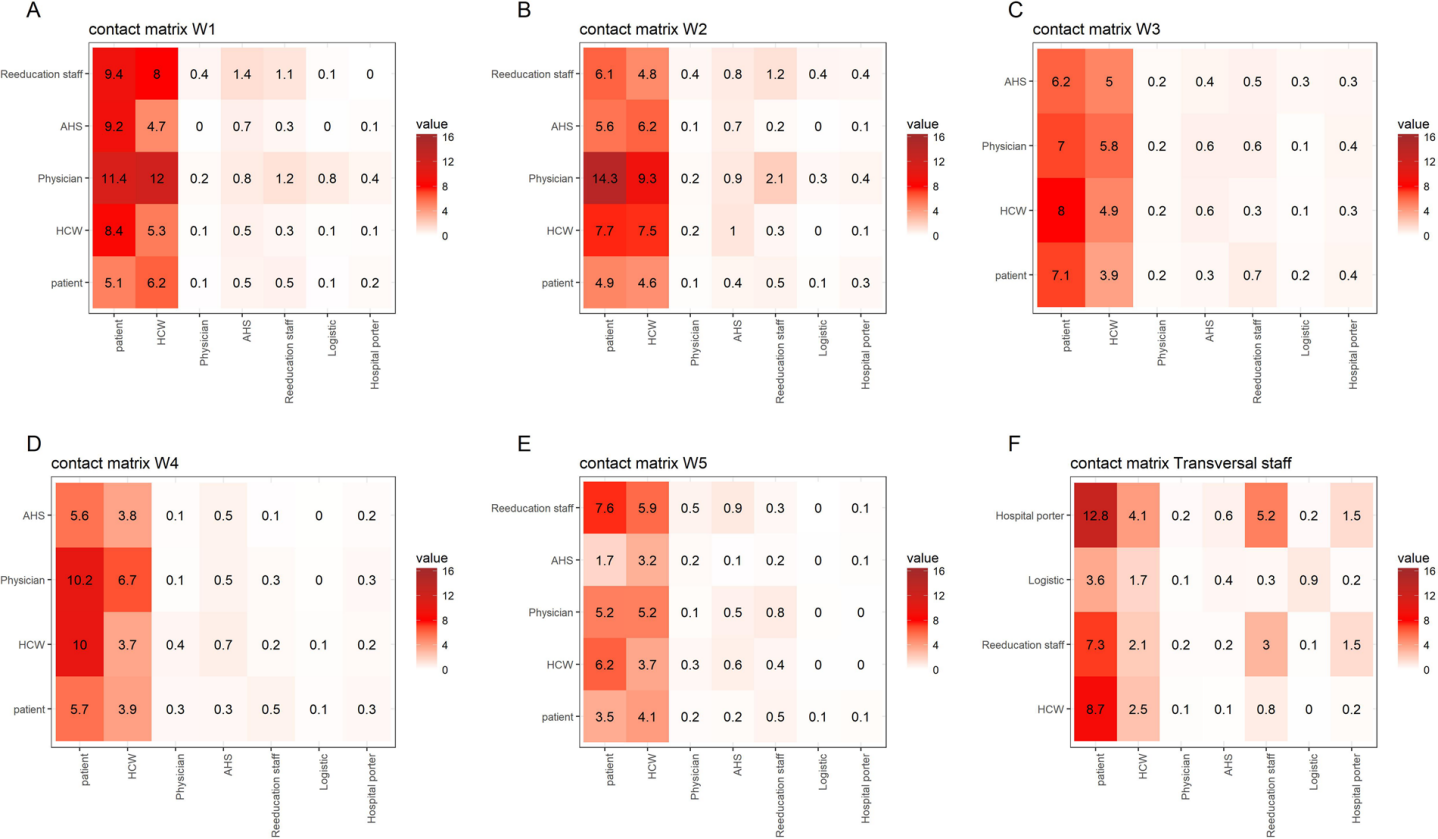
Ward-specific averaged daily distinct CPI frequency patterns between categories of individuals. Contact matrices are provided for (**A**) neurologic rehabilitation ward W1, (**B**) neurologic rehabilitation ward W2, (**C**) geriatric ward W3, (**D**) neurologic rehabilitation ward W4, (**E**) nutrition ward W5, and (**F**) individuals not attached to any ward (W6). Each cell represents the mean number of distinct individuals (see Supplementary Text S2) from a given category over the whole hospital (in columns) with whom someone from a category present in the ward (in rows) has a CPI.

Mixing patterns between staff groups and patients in each ward are depicted in Fig. 3 (in terms of daily distinct CPIs frequency) and 4 (in terms of cumulative duration of CPIs). Individuals attached to the neurologic rehabilitation wards W1 and W2 and transversal staff had the largest numbers of daily distinct CPIs with other categories; individuals from wards W1 and W2 also had the largest numbers of CPIs with hospital patients in general. Finally, patients from W3 and W1 had the largest numbers of CPIs (Fig. 3). Irrespective of the ward, the most frequent types of CPIs were hospital porter-patient, physician-patient and HCW-patient CPIs.

**Figure 4:**
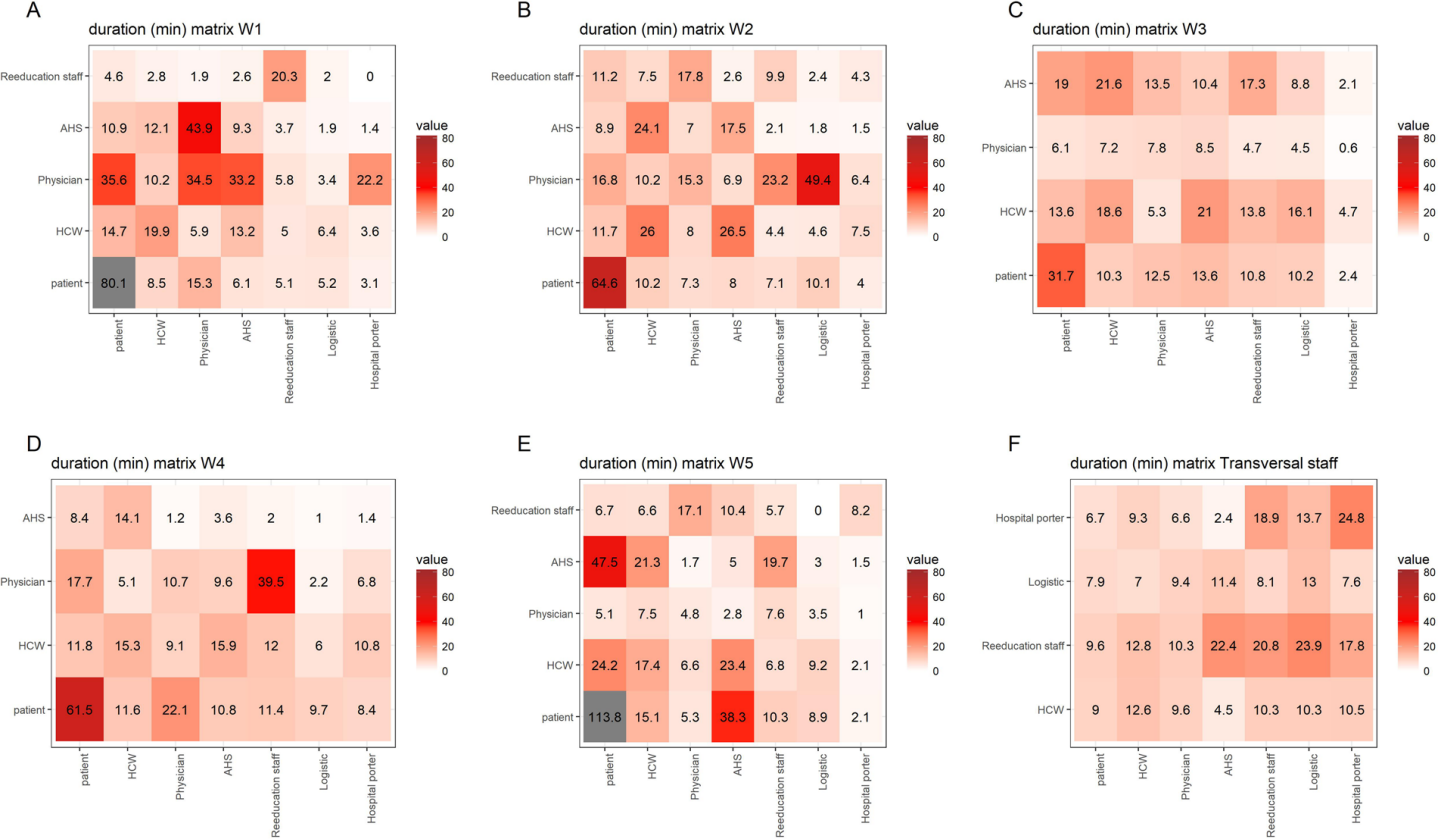
Ward-specific averaged CPI daily cumulative duration patterns between categories of individuals. Duration matrices are provided for (**A**) neurologic rehabilitation ward W1, (**B**) neurologic rehabilitation ward W2, (**C**) geriatric ward W3, (**D**) neurologic rehabilitation ward W4, (**E**) nutrition ward W5, and (**F**) individuals not attached to any ward (W6). Each cell represents the mean daily cumulative duration of CPIs between categories (see Supplementary Text S2) present in the corresponding ward (rows) and categories present in the whole hospital (columns).

The longest cumulative time spent in CPIs occurred for individuals attached to the geriatric rehabilitation ward W5 and neurologic rehabilitation ward W1 (Fig. 4). The average cumulative duration of patient-patient CPIs was long (69.4 min/day, 95% CI: 61.5–77.3), with a peak in geriatric ward W5, at nearly 2 hours/day (112.9 min/day). Physicians were the category which spent the most time with other categories, especially patients. To the contrary, the average cumulative CPI duration between HCWs and patients was short, at 13.5 min/day (95% CI: 11.4–15.7).

The detailed data on daily distinct CPIs frequency and cumulative duration of CPIs are provided for patients, as a function of reason for hospitalization, in Supplementary Table S1; and for hospital staff, as a function of category and ward, in Supplementary Table S2.

### Daily trends in CPIs

**Figure 5:**
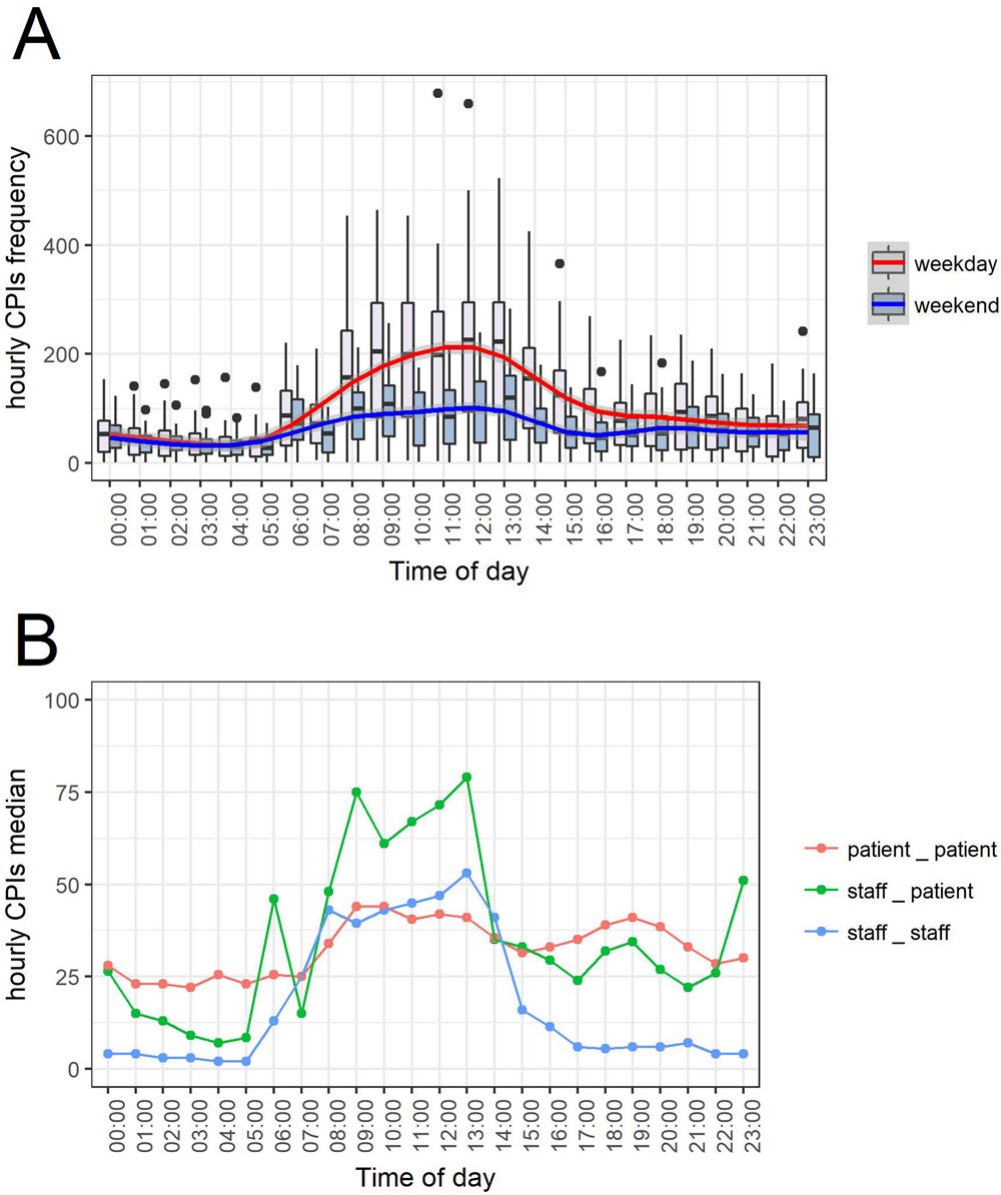
Time trends in CPIs over a 24-hour day. 5A: Boxplot of the distribution of hourly CPI frequencies for weekdays (pink box and red line) and weekend days (blue box and blue line). Blue and Red lines correspond to a GAM regression. 5B: median distinct hourly CPI frequency for patient-patient (red), staff-patient (green) and staff-staff (blue) CPIs for the study’s weeks.

Time trends in CPI patterns over a 24-hour day are reported in Fig. 5 and reflect the daily activities of individuals within the hospital. The majority of CPIs occurred in the morning, increasing from 5 a.m. to 11 a.m., with less CPIs during the weekends (Fig. 5A). Distinct hourly CPI frequency decreased during the afternoon, with a slight increase around 6–8 p.m. in patients (Fig. 5A,B). These trends varied according to the individuals in contact, with a less noticeable night/day difference in patient-patient hourly median CPI frequency (Fig. 5B).

### Factors associated with high daily contact frequency or cumulative duration among the staff

The statistical analyses for hospital staff were performed on 5,240 man-days. Staff category and days of the week were significantly associated with high distinct daily CPI frequency and high daily cumulative duration of CPIs (Table 1). Hospital porters (odds-ratio (OR): 10.81; 95% CI: 7.15–16.36) and physicians (OR: 2.99; 95% CI: 1.47–6.09) were at higher risk of having frequent CPIs than HCWs, unlike AHS (OR: 0.63; 95% CI: 0.46–0.88). Reeducation staff were at lower risk of having high cumulative duration of CPIs than HCWs (OR: 0.37, 95% CI: 0.22–0.63). Interestingly, Thursdays (OR: 1.38; 95% CI: 1.06–1.82) and Fridays (OR: 1.35, 5% CI: 1.04–1.75) were associated with higher distinct daily CPI frequency than Wednesdays, by opposition to Saturdays and Sundays during which CPIs were less frequent. Conversely, Sundays were associated with high cumulative duration of CPIs (OR: 1.88, 5% CI: 1.35–2.6).

**Table 1:** Factors associated with high daily distinct CPI frequency and high daily cumulative duration of CPIs among hospital staff, resulting from a mixed model with ward-specific random intercepts to account for within-ward and between-ward variations.

	*Factor*	*level*	*OR*	*CI 95%*	*p-value*
High daily distinct CPI frequency	Category (ref:HCW)	AHS	0.63	(0.46–0.88)**	1.10E-32
		Hospital porter	10.81	(7.15–16.36)***	
		Logistic	0.51	(0.25–1.03).	
		Physician	2.99	(1.47–6.09)**	
		Reeducation staff	1.24	(0.92–1.68)	
	days of week (ref: Wednesday)	Monday	0.86	(0.66–1.13)	2.30E-17
		Tuesday	1.00	(0.75–1.32)	
		Thursday	1.38	(1.06–1.82)*	
		Friday	1.35	(1.04–1.75)*	
		Saturday	0.65	(0.45–0.93)*	
		Sunday	0.25	(0.16–0.40)***	
High daily cumulative duration of CPIs	Category (ref: HCW)	AHS	1.07	(0.80–1.42)	5.50E-05
		Hospital porter	0.43	(0.16–1.20)	
		Logistic	0.81	(0.38–1.72)	
		Physician	0.00	(0.00–1.3e + 46)	
		Reeducation staff	0.37	(0.22–0.63)***	
	days of week (ref: Wednesday)	Monday	1.06	(0.77–1.46)	0.00075
		Tuesday	1.04	(0.75–1.46)	
		Thursday	0.87	(0.61–1.24)	
		Friday	1.14	(0.83–1.57)	
		Saturday	1.03	(0.71–1.51)	
		Sunday	1.88	(1.35–2.61)***	

Note: Observed level of the Wald test for each parameter: * < 0.05, ** < 0.01, *** < 0.001. OR: odds ratio.

### Factors associated with high daily contact frequency or cumulative duration among patients

The statistical analyses for the patients were performed on 7,219 man-days. Reasons for hospitalization, age and days of week were significantly associated with high distinct daily CPI frequency (Table 2). Post-operative patients (OR: 1.77, 95% CI: 1.15–2.73) were associated with high distinct daily CPI frequency, conversely to patients with nutritional issues. Three groups of age were associated with high distinct daily CPI frequency: the two youngest groups and the 60–70 years old group (OR: 1.3, 95% CI: 1.02–1.66), that was also associated with high daily cumulative duration of CPIs (OR: 1.42, 95% CI: 1.10–1.80). Similarly to what was observed for hospital staff, Thursdays (OR: 1.47, 95% CI: 1.14–1.90) and Fridays (OR: 1.52, 95% CI: 1.19–1.96) were associated with high distinct daily CPI frequency, conversely to Saturdays and Sundays. Saturdays (OR: 2.01, 95% CI: 1.52–2.7) and Sundays (OR: 3.39, 95% CI: 2.60–4.40) were associated with high daily cumulative duration of CPIs. Being a male patient (OR: 1.41, 95% CI: 1.12–1.5) was associated with high daily cumulative duration of CPIs.

**Table 2:** Factors associated with high daily distinct CPI frequency and high daily cumulative duration of CPIs among patients, resulting from a mixed model with ward-specific random intercepts to account for within-ward and between-ward variations.

	*Factor*	*level*	*OR*	*CI 95%*	*p-value*
High daily distinct CPI frequency	Reasons for hospitalization (ref: Orthopaedic)	Geriatric	0, 65	(0.22–1.95)	0,0017
		Neurology	0, 87	(0.61–1.25)	
		Nutrition	0, 64	(0.46–0.88)**	
		Post-operative	1, 77	(1.15–2.73)**	
	Age (ref: [50, 60))	[18, 30)	2, 27	(1.41–3.66)***	6,30E-06
		[30, 40)	1, 70	(1.29–2.21)***	
		[40, 50)	1, 08	(0.83–1.39)	
		[60, 70)	1, 30	(1.02–1.66)*	
		[70+]	0, 80	(0.58–1.09)	
	Gender (ref: Female)	Male	0, 86	(0.73–1.02).	0,084
	days of week (ref: Wednesday)	Monday	0, 82	(0.62–1.08)	1,10E-66
		Tuesday	0, 74	(0.56–0.98)*	
		Thursday	1, 47	(1.14–1.90)**	
		Friday	1, 52	(1.19–1.96)***	
		Saturday	0, 11	(0.06–0.19)***	
		Sunday	0, 16	(0.10–0.26)***	
High daily cumulative duration of CPIs	Reasons for hospitalization (ref: Orthopaedic)	Geriatric	2, 75	(0.87–8.75).	0,15
		Neurology	1, 31	(0.96–1.80).	
		Nutrition	0, 64	(0.19–2.15)	
		Post-operative	0, 91	(0.62–1.33)	
	Age (ref: [50, 60))	[18,30)	1, 04	(0.62–1.75)	8,70E-10
		[30,40)	1, 27	(0.97–1.66).	
		[40,50)	1, 18	(0.93–1.51)	
		[60,70)	1, 42	(1.10–1.82)**	
		[70+]	0, 41	(0.29–0.60)***	
	Gender (ref: Female)	Male	1, 31	(1.12–1.54)***	0,00083
	days of week (ref: Wednesday)	Monday	0, 92	(0.68–1.26)	1,30E-33
		Tuesday	1, 17	(0.87–1.58)	
		Thursday	1, 09	(0.81–1.49)	
		Friday	1, 11	(0.82–1.50)	
		Saturday	2, 01	(1.52–2.66)***	
		Sunday	3, 39	(2.60–4.41)***	

Note: Observed level of the Wald test for each parameter: * < 0.05, ** < 0.01, *** < 0.001. OR: odds ratio.

## Discussion

In this study, using RFID technology, the dynamics and heterogeneity of CPIs in a long-term care and rehabilitation hospital have been captured, providing detailed information on the pattern of interactions between different categories of hospital staff and patients, as well as between specific wards. In addition, factors associated with high distinct daily CPI frequency and cumulative duration have been identified.

### Comparison with the results of earlier studies

Some of our results are in accordance with previously published data on CPIs in hospital settings, while others are specific to the long-term setting. For instance, we found that physicians and HCWs (nurses and auxiliary nurses) had contacts with many distinct patients daily, in accordance with several earlier studies^9,10,14^. As expected, they spent a long time in contact with older patients and patients who need special cares, such as post-operative patient (Supplementary Table S4). However, in our setting, patients spent more time in contact with each other than with hospital staff, as opposed to what has been observed in acute-care settings^9^. Differences with contact patterns in acute-care settings may be due to specific activities taking place in rehabilitation hospitals, such as hairdressing and reeducation activities, which promote patient interactions with each other and with other kind of hospital staff such as reeducation staff and hospital porters. Indeed, this latter staff category was found to have high distinct CPI frequency in our study (Table 1). In addition, contacts were found to occur mostly during mornings, decreasing during the afternoon; a similar pattern was found in an earlier study^9^. We also showed a difference between weekdays and weekends. Moreover, the number of CPIs decreased along the week (Supplementary Fig. S1). CPIs were indeed longer during weekends, possibly related to social activities proposed by the LTCF. These time periods with high contact density may play an important part in pathogen spread dynamics.

### Implications of our results for infection control

In LTCF, by contrast to ICU, patients have longer stay and, as described above, share more time with other patients. This type of contact may lead to different patterns of transmission and therefore interventions proposed in ICU may not be adapted. Our results, which underline the high heterogeneity of contact patterns among individual categories and hospital wards, have potential implications for the design of future HAI control strategies in LTCF settings.

Ward-specific contact patterns can provide interesting information. During this study, the neurologic rehabilitation wards W1 and W2 were the most important wards in terms of distinct contact frequency (Fig. 3, Supplementary Tables S1 and S2). This is probably because these wards host patients who require special care and long treatments (Table 2), therefore needing repeated contacts with the staff. Moreover, patient-patient contacts were most frequent in ward W3, due to the fact that patients from this nutrition reeducation area were more mobile than neurologic or geriatric patients (Fig. 3, Supplementary Table S1) but their contacts were probably focused on patients as they were associated with lower risk of having high distinct daily CPI frequency. Another striking point concerns the geriatric ward W5 where high average contact duration was found for both patient-patient and staff-patient contacts, even though the total number of distinct CPIs remained low (Figs 3 and 4, Supplementary Tables S1 and S2). The information presented here could be used to propose interventions targeted at specific wards; however, this should also take into account ward-specific HAI prevalence. Interestingly, during the study period, neurologic rehabilitation ward W1 was the ward in which the incidence of ESBL-producing Enterobacteriaceae was highest and geriatric ward W5 was the ward in which the incidence of *Staphylococcus aureus* was the highest.

Because some categories of hospital staff were found to have contacts with large numbers of distinct patients daily, they may play an important role in pathogen spread dynamics. In particular, because they are not attached to a specific patient ward but may be in contact with individuals all over the hospital, hospital porters are potential “super-spreaders” if they do not comply with infection control recommendations such as hand hygiene; this may also be true of physicians who are in contact with patients from several wards^20,21^. Two different contact patterns are shown with these potential “super-spreaders”, with physicians having many daily distinct contacts with a long duration of contact with patients especially with older patients (Supplementary Table S4) and hospital porters having many daily distinct contacts with short duration. Interestingly, reeducation staff had many contacts with a long duration, especially with patients and on Fridays, due to a specific schedule (Supplementary Table S4). Future infection control strategies could be designed based on this data, for instance hand hygiene information and education interventions targeted at such “peripatetic” staff. Moreover, as mentioned earlier, a specificity of LTCF settings is the high frequency of contacts between patients, with patients spending a lot of time in contact with a limited number of other patients. This underlines the importance of including patients in infection control education interventions in LTCF settings.

### Implications of our results for mathematical modeling

Mathematical models represent a useful tool in the control of epidemics in healthcare settings^22^; however, a major issue for most published models is the lack of data on inter-individual contacts, leading to hypotheses such as homogeneous mixing, that are unrealistic in these highly clusterized settings characterized by small populations, where stochasticity impact is potentially strong. In this study, we provide a detailed description of the dynamic of contacts between individuals in a whole hospital that could help inform future computational models of the spread of healthcare-associated infections. In particular, we identify and characterize several distinct contact profiles, depending on patient characteristics, staff category or ward. This information can be used to build a complete realistic model of an LTCF. To our knowledge, this is the first study to report data at an entire hospital scale, rather than focusing on 1 or 2 wards^9–11^. All this information can be used to build a complete model of an LTCF. It is also the first report of data on inter-individual contacts within a long-term hospital. Because long-term care centers may represent a potential hotspot for the emergence and spread of healthcare-associated infections, in particular due to multi-resistant bacteria, this data has the potential to help better control the global dynamics of healthcare-associated infections^18,19^.

### Limitations of our study

This study had several strengths, including a very high participation rate, high spatial and temporal resolution, and long duration. However, it also presents several limitations which will be discussed.

First, although a good correlation between RFID data collection and direct observation has been reported^11^, this technology has some limits, in particular regarding its pertinence in terms of potential pathogen spread. In our study, every CPI at less than 1.5 m was recorded, without providing any information about the actual distance between individuals or potential physical contact. Hence, many brief interactions that do not actually present any potential for pathogen transmission may have been recorded. However, a recent analysis showed, using the same data, that *S. aureus* transmission within the hospital was consistent with contacts defined by these electronically collected CPIs^16^. More generally, the implications of measured CPIs in terms of infectious risk is bound to depend on pathogen-specific transmission modes - for instance, some of the CPIs may only play a part in epidemic dynamics if airborne transmission is possible.

Secondly, not only was CPI recording discontinued for 3 periods of two days over the 4-month study period due to battery changes; in some cases, battery failure interrupted record at random before the battery changing dates. This is a negative consequence of the long duration of our data collection using the RFID technology, as has been observed in earlier long-term RFID data collection^13^. However, even taking away the corresponding weeks, there was still enough statistical power to highlight dynamic contact patterns and to identify factors associated with high distinct contact frequency and duration. Furthermore, battery failure could be viewed as a random process. Missing data due to such events can therefore be considered missing at random and should not affect the differences in contact patterns that have been observed.

Finally, CPIs with visitors were not recorded in this study. While this may certainly limit our ability to fully understand the spread of pathogens that co-circulate in the community, the majority of contacts taking place within the hospital were still captured in our analysis.

## Conclusions

This unique study describes the dynamics of contacts between categories of individuals inside the hospital and through wards over a long period. Using such data to better inform contact patterns in mathematical models and simulators of pathogen transmission within hospitals is essential to improve our understanding of the spread of HAI (including antibiotic resistant bacteria) within hospitals and the realism of model predictions to propose optimized control measures.

## Methods

### Study setting

Administration and animation staff (including hairdressers and activity leaders) were not included in our analysis. Indeed, the data on animation staff was very limited, with only two individuals with recorded contacts, one of whom was only present for two days over the study period. Moreover, administration staff have limited contact with other staff and patients, with very little risk of HAI transmission. Likewise, patients in Persistent Vegetative State (PVS) were not include in our study.

### Data collection

The overall participation rate was 90.1%. All participating staff and patients wore a small wireless sensor recording Close-Proximity Interactions (CPIs, typically at less than 1.5 m) over their entire presence in the hospital. Because RFID devices only exchange packets when individuals are face-to-face, as the human body acts as a shield at the frequency used for communication, only front-facing CPIs were recorded. CPIs were recorded every 30 seconds, along with the time, date and anonymous identifiers of the receiving and transmitting sensors. Over the 6-month duration of the study, sensor batteries had to be replaced on 3 occasions, during which recording was discontinued for 2 days.

### Descriptive analysis of CPIs

The patterns of CPIs within the hospital were analyzed using several quantities related to frequency and duration of contacts.

At the daily scale, two main quantities were computed:

- ***Number of daily distinct CPIs of a given individual*** was calculated as the total number of different individuals met during a day, to depict the **frequency** of distinct CPIs
- ***Daily cumulative duration of CPIs of two individuals*** was calculated as the cumulative duration of CPI spent with each other over one day, as a proxy of the **duration** of contacts.
  To characterize group-specific CPI patterns, we averaged these quantities over all individuals from each category (staff groups or patients); mathematical definitions are provided in Supplementary text S1.
  Mixing patterns between different staff and patients categories were analysed. To do so, we built matrices depicting the CPIs of patients and each staff group from the ward with other patients and staff from the whole hospital. For each ward, we defined:
- The contact matrix, which depicts the averaged daily distinct CPI frequency for each category of the ward,
- The duration matrix, which depicts the averaged CPI daily cumulative duration for each category of the ward.

For instance, one matrix cell may represent the mean number of daily distinct CPIs (or the mean daily cumulative duration of CPIs) of one staff group (or of patient) present in the ward with individuals from another staff group (or with patients) present in the entire hospital, irrespective of the ward (see Supplementary Text S2 for more details).

Finally, time changes in CPI patterns at the hospital scale over a typical 24-hour day were analysed. We calculated the distribution of the **hourly** number of recorded CPIs (hourly CPIs frequency) over the study period (excluding the three weeks during which battery change occurred). This was done separately for weekdays and for weekends. In order to observe pattern differences between patient-patient, staff-staff and staff-patient CPIs, time changes for the three types of contacts were also studied specifically. This was achieved by comparing the medians of the three types of contacts’ hourly CPIs distribution.

### Statistical analysis

To determine which factors were associated with high level of CPI frequency and cumulative duration, two sets of statistical analyses were performed for patients and staff separately. For each patient or hospital staff, contact frequency (average number of daily distinct CPIs) and cumulative duration (average number of daily cumulative duration of CPIs) were calculated (Supplementary Text S3). Daily contact frequency and daily cumulative duration were first transformed into discrete variables with 2 classes (low/high), using the mean of these two variables plus 1 standard deviation (sd) as threshold. Other thresholds (mean, mean + 2 sd, mean + 3 sd) were also investigated as a sensitivity analysis (Supplementary Table S3). These outcomes were modeled as a function of, respectively, staff category (for the staff) or reasons for hospitalization, age and gender (for patients). We used generalized linear mixed-effects models (GLMM) in order to account for the heterogeneity through a statistical parameter representing inter-ward variation and adjust for patient or hospital staff characteristics (Supplementary Text S4). Each factor associated p-value of the GLMM was calculated using the likelihood ratio test of the “mixed” function from the R package afex^23^. All confidence intervals of distributions were based on Student’s t-Test. All analyses were performed with R (version 3.2.3)^24^.

### Ethics

The study obtained all authorizations in accordance with French regulations regarding medical research and information processing. All French IRB-equivalent agencies accorded the i-Bird program official approval (CPP 08061; Afssaps 2008-A01284-51; CCTIRS 08.533; CNIL AT/YPA/SV/SN/GDP/AR091118 N°909036). Signed consent by patients and staff was not required according to the French Ethics Committee to which the project was submitted.

## Electronic supplementary material

Supplementary material
