# Author Correction: Measuring dynamic social contacts in a rehabilitation hospital: effect of wards, patient and staff characteristics

**DOI:** 10.1038/s41598-020-57824-y

**Published:** 2020-01-30

**Authors:** Audrey Duval, Thomas Obadia, Lucie Martinet, Pierre-Yves Boëlle, Eric Fleury, Didier Guillemot, Lulla Opatowski, Laura Temime, I-Bird study group, Anne Sophie Alvarez, Audrey Baraffe, Mariano Beiró, Inga Bertucci, Camille Cyncynatus, Florence Dannet, Marie Laure Delaby, Pierre Denys, Matthieu Domenech de Cellès, Antoine Fraboulet, Jean-Louis Gaillard, Jean-Louis Herrmann, Boris Labrador, Jennifer Lasley, Christine Lawrence, Judith Legrand, Odile Le Minor, Caroline Ligier, Karine Mignon, Catherine Sacleux, Jérôme Salomon, Marie Perard, Laure Petit, Laeticia Remy, Anne Thiebaut, Damien Thomas, Philippe Tronchet, Isabelle Villain

**Affiliations:** 1 Biostatistics, Biomathematics, Pharmacoepidemiology and Infectious Diseases (B2PHI), Inserm, UVSQ, Institut Pasteur, Université Paris-Saclay, Paris, France; 2 Institut Pasteur – Bioinformatics and Biostatistics Hub – C3BI, USR, 3756 IP CNRS Paris, France; 3 Malaria Parasites & Hosts Unit, Department of Parasites & Insect Vectors, Institut Pasteur, Paris, France; 4 ENS de Lyon, DANTE/INRIA, LIP UMR CNRS 5668 Université de Lyon, Lyon, France; 5 Sorbonne Universités, UPMC Univ Paris 06, UMR_S 1136, Institut Pierre Louis d’Epidémiologie et de Santé Publique, Paris, France; 6 ENS de Lyon, Université de Lyon, Laboratoire de l’Informatique du Parallélisme (UMR CNRS 5668- ENS de Lyon-UCB Lyon 1), IXXI Rhône Alpes Complex Systems Institute, Lyon, France; 7 INSERM 1181 Biostatistics, Biomathematics, Pharmacoepidemiology and Infectious Diseases (B2PHI), Institut Pasteur, B2PHI Paris, France; 8 Université de Versailles Saint-Quentin, UMR 1181, B2PHI Montigny-Le-Bretonneux, France; 9 AP-HP, Raymond-Poincaré Hospital, Garche, France; 10 Laboratoire MESuRS, Conservatoire national des Arts et Métiers, Paris, France; 11 Unité PACRI, Institut Pasteur, Conservatoire national des Arts et Métiers, Paris, France; 12 AP-HP, Paris, France; 13 Universidad de Buenos Aires, Buenos Aires, Argentina; 14 AbAg, Chilly-Mazarin, France; 15 Insa, Lyon, France; 16 Inserm, Paris, France; 17 Univ Paris-Sud, UMR 0320/UMR8120 Génétique Quantitative et Evolution Le Moulon, Université Paris-Saclay, F-91190 Gif-sur-Yvette, France; 18 Institut Pasteur, Paris, France

Correction to: *Scientific Reports* 10.1038/s41598-018-20008-w, published online 26 January 2018

The Supplementary Material file that accompanies this Article contains an error in equations 3 and 5 and the paragraphs that follow them. Equation 3 and the paragraph below it:

$${\forall }_{c,c}DCD(c)=\frac{1}{{N}_{c}}{\sum }_{i{\epsilon }c}\frac{1}{{D}_{i}}{\sum }_{t=1}^{{D}_{i}}{\sum }_{j\in H(t)}DCD(i,j,t)$$

Where *c* is the category (eg. HCW, patients, etc.), *N*_*c*_ is the number of individuals *i* belonging to category *c*, *D*_*i*_ the number of days of presence of individual *i* in the hospital, H(t) is the ensemble of all individuals present in the hospital on day *t*, and *DCD(i,j,t)* the cumulative duration of CPIs on day *t* between individuals *i* and *j*.

should read:

$${\forall }_{c,c}DCD(c)=\frac{1}{{N}_{c}}{\sum }_{i{\epsilon }c}\frac{1}{H}{\sum }_{j{\epsilon }H}\frac{1}{{D}_{i,j}}{\sum }_{t{\epsilon }{D}_{i,j}}DCD(i,j,t)$$

Where *c* is the category (eg. HCW, patients, etc.), *N*_*c*_ is the number of individuals *i* belonging to category *c*, *D*_*i,j*_ the number of days of contact between *i* and *j* in the hospital, *H* is the ensemble of all individuals present in the hospital, and *DCD(i,j,t)* the cumulative duration of CPIs on day *t* between individuals *i* and *j*.

Equation 5 and the paragraph below it:

$$\forall (w,{c}_{1},{c}_{2}),wDCD({c}_{1},{c}_{2})=\frac{1}{{N}_{w,c1}}{\sum }_{i=1}^{{N}_{w,c1}}\frac{1}{{D}_{i}}{\sum }_{t=1}^{{D}_{i}}{\sum }_{j=1}^{{N}_{c2}}DCD(i,j,t)$$

Where *c*_*1*_ and *c*_*2*_ are two categories, *w* is the ward number, *N*_*w,c1*_ is the number of individuals *s* belonging to category *c*_*1*_ (staff categories or patients), an *D*_*i*_ is the number of days of presence of individual *i* in the hospital, *N*_*c2*_ is the number of individuals from category *c*_*2*_ in the hospital, and *DCD(i,j,t)* is the *daily cumulative duration of CPIs* of two individuals *i* and *j* on day *t*.

should read:

$$\forall (w,{c}_{1},{c}_{2}),wDCD({c}_{1},{c}_{2})=\frac{1}{{N}_{w,c1}}{\sum }_{i=1}^{{N}_{w,c1}}\frac{1}{{N}_{c2}}{\sum }_{j=1}^{{N}_{c2}}\frac{1}{{D}_{i,j}}{\sum }_{j=1}^{{D}_{i,j}}DCD(i,j,t)$$

Where c_1_ and c_2_ are two categories, *w* is the ward number, *N*_*w,c1*_ is the number of individuals *s* belonging to category *c*_*1*_ (staff categories or patients), and *D*_*i,j*_ is the number of days of contact between *i* and *j* in the hospital, *N*_*c2*_ is the number of individuals from category *c*_*2*_ in the hospital, and *DCD(i,j,t)* is the *daily cumulative duration of CPIs* of two individuals *i* and *j* on day *t*.

