# Supplementary material for: Measuring dynamic social contacts in a rehabilitation hospital: effect of wards, patient and staff characteristics
Source: Sci Rep. 2018 Jan 26;8:1686. doi: 10.1038/s41598-018-20008-w (PMC5786108; doi:10.1038/s41598-018-20008-w)
Supplement: Supplementary file 1 — Supplementary material [file 41598_2018_20008_MOESM1_ESM.pdf]

Measuring dynamic social contacts in a rehabilitation hospital: effect of wards, patient and staff characteristics.

Audrey Duval*[1], Thomas Obadia[2, 3], Lucie Martinet[4], Pierre-Yves Boëlle[5], Eric Fleury[6], Didier Guillemot**[7, 8, 9], Lulla Opatowski**[1], Laura Temime**[10, 11], on behalf of I-Bird study group

** Equally contributed

Correspondence to lulla.opatowski@pasteur.fr

1 Biostatistics, Biomathematics, Pharmacoepidemiology and Infectious Diseases (B2PHI), Inserm, UVSQ, Institut Pasteur, Université Paris-Saclay, France

2 Institut Pasteur – Bioinformatics and Biostatistics Hub – C3BI, USR 3756 IP CNRS – Paris, France

3 Malaria Parasites & Hosts Unit, Department of Parasites & Insect Vectors, Institut Pasteur, Paris, France

4 ENS de Lyon, DANTE/INRIA, LIP UMR CNRS 5668 Université de Lyon, Lyon, France

5 Sorbonne Universités, UPMC Univ Paris 06, UMR_S 1136, Institut Pierre Louis d'Epidémiologie et de Santé Publique, Paris, France

6 ENS de Lyon, Université de Lyon, Laboratoire de l'Informatique du Parallélisme (UMR CNRS 5668-ENS de Lyon-UCB Lyon 1), IXXI Rhône Alpes Complex Systems Institute, Lyon, France

7 INSERM 1181 Biostatistics, Biomathematics, Pharmacoepidemiology and Infectious Diseases (B2PHI), Institut Pasteur, B2PHI, Paris, France

8 Université de Versailles Saint-Quentin, UMR 1181, B2PHI, Montigny-Le-Bretonneux, France

9 AP-HP, Raymond-Poincaré Hospital, Garche, France

10 Laboratoire MESuRS, Conservatoire national des Arts et Métiers, Paris, France

11 Unité PACRI, Institut Pasteur, Conservatoire national des Arts et Métiers, Paris, France

Supplementary material

Supplementary Table S1: mean (sd) of the number of daily distinct CPIs and the daily cumulative CPI duration in minutes among patients

		CPIs frequency mean (sd)	CPIs duration (minutes) mean (sd)
ward	W1	12.74 (4.56)	32.58 (18.68)
	W2	10.93 (5.04)	30.27 (20.12)
	W3	12.9 (7.34)	20.9 (19.02)
	W4	11.14 (5.9)	32.26 (19.95)
	W5	8.58 (5.13)	47.16 (33.67)
Reason for hospitalization	Neurology	11.85 (4.94)	31.36 (19.08)
	Orthopedic	10.73 (5.46)	26.11 (19.29)
	Nutrition	12.89 (7.59)	22.56 (21.71)
	Post-operative	14.71 (9.16)	34.53 (16.39)
	Geriatric	8.62 (5.16)	47.19 (33.92)

Supplementary Table S2: mean (sd) of the number of daily distinct CPIs and the daily cumulative CPI duration in minutes among hospital staff

		CPIs frequency mean (sd)	CPIs duration (minutes) mean (sd)
Hospital staff	Hospital porter	24.58 (13.4)	8.96 (4.41)
	Physician	21.32 (7.02)	13.2 (6.76)
	HCW	14.29 (5.29)	14.94 (8.28)
	Reeducation staff	15.03 (6.04)	11.6 (9.62)
	AHS	12 (6.22)	15.71 (9.52)
	Logistic	7.23 (3.86)	8.07 (9.43)
ward	W1	15.34 (6.07)	14.7 (9.64)
	W2	16.81 (6.81)	17.21 (6.13)
	W3	14.6 (3.31)	15.1 (7.54)
	W4	14.91 (4.47)	12.84 (3.72)
	W5	10.98 (3.97)	18.8 (11.85)
	Transversal staff	12.73 (7.18)	11.67 (15.57)

Supplementary Text S1: Calculation of individual-specific and category-specific daily distinct CPIs and cumulative duration of CPIs

The ***number of daily distinct CPIs*** (DDC) for individual *i* on day *t* was calculated according to the following formula:

(1) $DDC(i,t) = \sum_{j \in H(t)} I(i,j,t)$

Where *t* is a day of presence of individual *i* in the hospital, and *H(t)* is the ensemble of all individuals present in the hospital on day *t* and *I(i,j,t)* is defined by:

$$I(i,j,t) = \begin{cases} 0, & \text{if no CPI recorded between } i \text{ and } j \text{ on day t} \\ 1, & \text{if at least one recorded CPI between } i \text{ and } j \text{ on day t} \end{cases}$$

The ***daily cumulative duration of CPIs*** of two individuals *i* and *j* on day *t* is noted *DCD(i,j,t)* and is calculated as the sum of all CPI durations shared by *i* and *j* on day *t*.

The ***number of category-specific daily distinct CPIs*** (cDDC) depicted in Fig. 2A was calculated according to the following formula:

$$(2)\ \forall\, c, cDDC(c) = \frac{1}{N_c}\sum_{i \in c}\frac{1}{D_i}\sum_{t=1}^{D_i} DDC(i,t)$$

Where *c* is the category (eg. HCW, patients, etc.), N_c is the number of individuals *i* belonging to category *c*, D_i the number of days of presence of individual *i* in the hospital and *DDC(i,t)*, the number of daily distinct CPIs on day *t* for individual *i*.

The ***category-specific cumulative duration of CPIs*** (cDCD) depicted in Fig. 2B was calculated as follows:

$$(3)\ \forall\, c, cDCD(c) = \frac{1}{N_c}\sum_{i \in c}\frac{1}{D_i}\sum_{t=1}^{D_i}\sum_{j \in H(t)} DCD(i,j,t)$$

Where *c* is the category (eg. HCW, patients, etc.), N_c is the number of individuals *i* belonging to category *c*, D_i the number of days of presence of individual *i* in the hospital, H(t) is the ensemble of all individuals present in the hospital on day *t*, and *DCD(i,j,t)* the cumulative duration of CPIs on day *t* between individuals *i* and *j*.

Supplementary Text S2: Calculation of contact frequency and contact duration matrices

For each ward *w*, the contact matrix depicts the CPI frequency for all individuals of the ward according to their category. The values depicted in Fig. 3 are calculated as follows:

$$(4)\ \forall\, (w, c_1, c_2), wDDC(c_1, c_2) = \frac{1}{N_{w,c1}}\sum_{i=1}^{N_{w,c1}}\frac{1}{D_i}\sum_{t=1}^{D_i}\sum_{j=1}^{N_{c2}} I(i,j,t)$$

Where c_1 and c_2 are two categories, *w* is the ward number, $N_{w,c1}$ is the number of individuals *s* belonging to category c_1 (staff categories or patients), an D_i is the number of days of presence of individual *i* in the hospital, N_{c2} is the number of individuals from category c_2 in the hospital, and *I(i,j,t)* equals 1 if at least one contact was recorded between *i* and *j* on day *t* and 0 otherwise.

For each ward *w*, the contact duration for a given category and a given ward depicted in Fig. 4 was calculated:

$$(5)\ \forall\, (w, c_1, c_2), wDCD(c_1, c_2) = \frac{1}{N_{w,c1}}\sum_{i=1}^{N_{w,c1}}\frac{1}{D_i}\sum_{t=1}^{D_i}\sum_{j=1}^{N_{c2}} DCD(i,j,t)$$

Where c_1 and c_2 are two categories, *w* is the ward number, $N_{w,c1}$ is the number of individuals *s* belonging to category c_1 (staff categories or patients), an D_i is the number of days of presence of

individual i in the hospital, N_{c2} is the number of individuals from category c_2 in the hospital, and DCD(i,j,t) is the *daily cumulative duration of CPIs* of two individuals i and j on day t.

Supplementary Text S3: Calculation of the threshold used for the GLMM

$$(6)\ M_w = \frac{1}{N}\sum_{p=1}^{N}\frac{1}{Nd_p}\sum_{k=1}^{Nd_p} C_k^p$$

Where N individuals included in the study. Nd_p the number of days for which contacts were recorded for individual present (indicator or presence in the hospital), C_k^p is the number of distinct CPI (or cumulative duration with other individual) recorded for individual p on day k.

Supplementary Text S4: description of the generalized linear mixed-effects models (GLMM) equations

The GLMM model of the high daily contact frequency and high daily cumulative duration among hospital staff was calculated:

$$(7)\ Y_{among\ hospital\ staff} = (\beta_0 + \beta_{ward}) + \beta_1 Category + \beta_2 days\ of\ week$$

Where $Y_{among\ hospital\ staff}$ represent the high daily contact frequency or the high daily cumulative duration. β_{ward} represents the ward specific random intercepts, β_1 and β_2 the fixed effect of the category and days of week as follows.

The GLMM model of the high daily contact frequency and high daily cumulative duration among patient was calculated as:

$$(8)\ Y_{among\ patient} = (\beta_0 + \beta_{ward}) + \beta_1 Reasons\ for\ hospitalization\ + \beta_2 Age + \beta_3 Gender + \beta_4 days\ of\ week$$

Where $Y_{among\ patient}$ represent the high daily contact frequency or the high daily cumulative duration. β_{ward} represents the ward specific random intercepts and β_1, β_2, β_3 and β_4 the fixed effect of the reasons for hospitalization, age, gender and days of week.

Supplementary Table S3: sensitivity analysis of the generalized linear mixed-effects models with different thresholds (mean, mean + 1 standard deviation (SD), mean + 2 SD and mean + 3SD). Factors associated with high daily distinct CPI frequency and high daily cumulative duration of CPIs among hospital staff or patients, resulting from a mixed model with ward-specific random intercepts to account for within-ward and between-ward variations.

	Factor	*level*	*OR*	*CI 95%*	*p-value*
High daily distinct CPI frequency among hospital staff (mean threshold)	Category (ref :HCW)	AHS	0.40	(0.32 - 0.50) ***	9,00E-39
		Hospital porter	4.54	(2.85 - 7.22) ***	
		Logistic	0.23	(0.15 - 0.37) ***	

		Physician	1.32	(0.66 - 2.64)	
		Reeducation staff	0.81	(0.66 - 1.00)	
	days of week (ref: Wednesday)	Monday	0.91	(0.75 - 1.10)	1.20E-08
		Tuesday	0.94	(0.77 - 1.15)	
		Thursday	1.15	(0.94 - 1.41)	
		Friday	1.00	(0.82 - 1.22)	
		Saturday	0.83	(0.66 - 1.05)	
		Sunday	0.53	(0.42 - 0.67) ***	
High daily cumulative duration of CPIs among hospital staff (mean threshold)	Category (ref :HCW)	AHS	1.25	(1.01 - 1.54) *	1.40E-06
		Hospital porter	0.60	(0.33 - 1.10)	
		Logistic	0.59	(0.33 - 1.05)	
		Physician	0.20	(0.06 - 0.68) **	
		Reeducation staff	0.52	(0.38 - 0.71) ***	
	days of week (ref: Wednesday)	Monday	1.03	(0.82 - 1.30)	0.4
		Tuesday	1.09	(0.85 - 1.38)	
		Thursday	1.18	(0.92 - 1.50)	
		Friday	1.15	(0.91 - 1.45)	
		Saturday	1.07	(0.81 - 1.40)	
		Sunday	1.32	(1.02 - 1.71) *	
High daily distinct CPI frequency among patients (mean threshold)	Reasons for hospitalization (ref: Orthopaedic)	Geriatric	5.62	(0.78 - 40.48)	0.012
		Neurology	0.86	(0.68 - 1.08)	
		Nutrition	0.74	(0.57 - 0.95) *	
		Post-operative	0.92	(0.69 - 1.22)	
	Age (ref: [50,60))	[18,30)	1.75	(1.22 - 2.50) **	0.00043
		[30,40)	1.25	(1.04 - 1.51) *	
		[40,50)	0.91	(0.77 - 1.07)	
		[60,70)	1.09	(0.92 - 1.28)	
		[70+]	1.25	(1.03 - 1.52) *	
	Gender (ref: Female)	Male	0.76	(0.68 - 0.85) ***	1.80E-06
	days of week (ref:	Monday	1.07	(0.89 - 1.27)	9,00E-164
		Tuesday	0.71	(0.59 - 0.86) ***	

	Wednesday)	Thursday	1.08	(0.90 - 1.29)	
		Friday	1.67	(0.98 - 1.39)	
		Saturday	0.20	(0.16 - 0.25) ***	
		Sunday	0.15	(0.12 - 0.19) ***	
High daily cumulative duration of CPIs among patients (mean threshold)	Reasons for hospitalization (ref: Orthopaedic)	Geriatric	1.20	(0.59 - 2.46)	0.0079
		Neurology	1.42	(1.13 - 1.78) **	
		Nutrition	0.69	(0.45 - 1.07)	
		Post-operative	1.06	(0.81 - 1.38)	
	Age (ref: [50,60))	[18,30)	0.70	(0.48 - 1.01)	2.60E-07
		[30,40)	0.97	(0.80 - 1.17)	
		[40,50)	1.13	(0.95 - 1.33)	
		[60,70)	1.00	(0.84 - 1.20)	
		[70+]	0.58	(0.47 - 0.71) ***	
	Gender (ref: Female)	Male	1.07	(0.96 - 1.20)	0.2
	days of week (ref: Wednesday)	Monday	1.13	(0.93 - 1.37)	4.20E-33
		Tuesday	1.16	(0.95 - 1.41)	
		Thursday	1.15	(0.94 - 1.40)	
		Friday	1.20	(0.98 – 1.45)	
		Saturday	1.98	(1.63 - 2.41) ***	
		Sunday	2.59	(2.14 - 3.13) ***	
High daily distinct CPI frequency among hospital staff (mean plus one SD threshold)	Category (ref :HCW)	AHS	0.63	(0.46 - 0.88) **	1.10E-32
		Hospital porter	10.81	(7.15 - 16.36) ***	
		Logistic	0.51	(0.25 - 1.03)	
		Physician	2.99	(1.47 - 6.09) **	
		Reeducation staff	1.24	(0.92 - 1.68)	
	days of week (ref: Wednesday)	Monday	0.86	(0.66 - 1.13)	2.30E-17
		Tuesday	1.00	(0.75 - 1.32)	
		Thursday	1.38	(1.06 - 1.82) *	
		Friday	1.35	(1.04 - 1.75) *	
		Saturday	0.65	(0.45 - 0.93) *	
		Sunday	0.25	(0.16 - 0.40) ***	

High daily cumulative duration of CPIs among hospital staff (mean plus one SD threshold)	Category (ref :HCW)	AHS	1.07	(0.80 - 1.42)	5.50E-05
		Hospital porter	0.43	(0.16 - 1.20)	
		Logistic	0.81	(0.38 - 1.72)	
		Physician	0.00	(0.00 - 1.3e+46)	
		Reeducation staff	0.37	(0.22 - 0.63) ***	
	days of week (ref: Wednesday)	Monday	1.06	(0.77 - 1.46)	0.00075
		Tuesday	1.04	(0.75 - 1.46)	
		Thursday	0.87	(0.61 - 1.24)	
		Friday	1.14	(0.83 - 1.57)	
		Saturday	1.03	(0.71 - 1.5)	
		Sunday	1.88	(1.35 - 2.61) ***	
High daily distinct CPI frequency among patients (mean plus one SD threshold)	Reasons for hospitalization (ref: Orthopaedic)	Geriatric	0.65	(0.22 - 1.95)	0.0017
		Neurology	0.87	(0.61 - 1.25)	
		Nutrition	0.64	(0.46 - 0.88) **	
		Post-operative	1.77	(1.15 - 2.73) **	
	Age (ref: [50,60))	[18,30)	2.27	(1.41 - 3.66) ***	6.30E-06
		[30,40)	1.70	(1.29 - 2.21) ***	
		[40,50)	1.08	(0.83 - 1.39)	
		[60,70)	1.30	(1.02 - 1.66) *	
		[70+]	0.80	(0.58 - 1.09)	
	Gender (ref: Female)	Male	0.86	(0.73 - 1.02) .	0.084
	days of week (ref: Wednesday)	Monday	0.82	(0.62 - 1.08)	1.10E-66
		Tuesday	0.74	(0.56 - 0.98) *	
		Thursday	1.47	(1.14 - 1.90) **	
		Friday	1.52	(1.19 - 1.96) ***	
		Saturday	0.11	(0.06 - 0.19) ***	
		Sunday	0.16	(0.10 - 0.26) ***	
High daily cumulative duration of CPIs among patients (mean plus one SD threshold)	Reasons for hospitalization (ref:	Geriatric	2.75	(0.87 - 8.75)	0.15
		Neurology	1.31	(0.96 - 1.80)	
		Nutrition	0.64	(0.19 - 2.15)	
		Post-operative	0.91	(0.62 - 1.33)	

	Orthopaedic)				
	Age (ref: [50,60))	[18,30)	1.04	(0.62 - 1.75)	8.70E-10
		[30,40)	1.27	(0.97 - 1.66) .	
		[40,50)	1.18	(0.93 - 1.51)	
		[60,70)	1.42	(1.10 - 1.82) **	
		[70+]	0.41	(0.29 - 0.60) ***	
	Gender (ref: Female)	Male	1.31	(1.12 - 1.54) ***	0.00083
	days of week (ref: Wednesday)	Monday	0.92	(0.68 - 1.26)	1.30E-33
		Tuesday	1.17	(0.87 - 1.58)	
		Thursday	1.09	(0.81 - 1.49)	
		Friday	1.11	(0.82 - 1.50)	
		Saturday	2.01	(1.52 - 2.7) ***	
		Sunday	3.39	(2.60 - 4.41) ***	
High daily distinct CPI frequency among hospital staff (mean plus two SD threshold)	Category (ref :HCW)	AHS	0.59	(0.23 - 1.23)	3.80E-29
		Hospital porter	20.70	(11.76 - 36.44) ***	
		Logistic	0.55	(0.13 - 2.38)	
		Physician	8.74	(3.75 - 20.37) ***	
		Reeducation staff	1.21	(0.66 - 2.21)	
	days of week (ref: Wednesday)	Monday	1.46	(0.84 - 2.54)	1,00E-15
		Tuesday	0.74	(0.38 - 1.44)	
		Thursday	2.30	(1.33 - 3.97) **	
		Friday	3.21	(1.92 - 5.35) ***	
		Saturday	0.30	(0.08 - 1.00)	
		Sunday	0.09	(0.01 - 0.65) *	
High daily cumulative duration of CPIs among hospital staff (mean plus two SD threshold)	Category (ref :HCW)	AHS	0.95	(0.62 - 1.45)	2.80E-06
		Hospital porter	0.18	(0.02 - 1.33)	
		Logistic	1.02	(0.43 - 2.44)	
		Physician	0.00	(0.00 - Inf)	
		Reeducation staff	0.09	(0.02 - 0.35) ***	

	days of week (ref: Wednesday)	Monday	0.83	(0.51 - 1.35)	0.00063
		Tuesday	1.07	(0.66 - 1.74)	
		Thursday	0.93	(0.56 - 1.54)	
		Friday	1.12	(0.71 - 1.79)	
		Saturday	1.33	(0.80 - 2.21)	
		Sunday	2.24	(1.44 - 3.49) ***	
High daily distinct CPI frequency among patients (mean plus two SD threshold)	Reasons for hospitalization (ref: Orthopaedic)	Geriatric	0.65	(0.29 - 1.47)	0.047
		Neurology	1.02	(0.63 - 1.63)	
		Nutrition	1.24	(0.72 - 2.13)	
		Post-operative	2.33	(1.27 - 4.25) **	
	Age (ref: [50,60))	[18,30)	2.10	(1.04 - 4.25) *	0.074
		[30,40)	1.37	(0.88 - 2.14)	
		[40,50)	0.88	(0.57 - 1.35)	
		[60,70)	1.00	(0.65 - 1.54)	
		[70+]	0.68	(0.39 - 1.19)	
	Gender (ref: Female)	Male	0.86	(0.64 - 1.15)	0.31
	days of week (ref: Wednesday)	Monday	1.22	(0.69 - 2.13)	2,00E-39
		Tuesday	1.09	(0.61 - 1.94)	
		Thursday	3.28	(2.02 - 5.31) ***	
		Friday	3.14	(1.94 - 5.08) ***	
		Saturday	0.00	(0.00 - Inf)	
		Sunday	0.04	(0.01 - 0.31) **	
High daily cumulative duration of CPIs among patients (mean plus two SD threshold)	Reasons for hospitalization (ref: Orthopaedic)	Geriatric	9.35	(1.54 - 56.67) *	0.065
		Neurology	0.89	(0.51 - 1.55)	
		Nutrition	0.25	(0.04 - 1.57)	
		Post-operative	0.82	(0.41 - 1.60)	
	Age (ref: [50,60))	[18,30)	0.81	(0.28 - 2.30)	6.30E-08
		[30,40)	2.12	(1.44 - 3.12) ***	
		[40,50)	1.16	(0.78 - 1.74)	
		[60,70)	1.57	(1.07 - 2.29) *	
		[70+]	0.38	(0.21 - 0.69) **	

	Gender (ref: Female)	Male	1.47	(1.14 - 1.88) **	0.0026
	days of week (ref: Wednesday)	Monday	1.09	(0.65 - 1.85)	3.70E-15
		Tuesday	1.66	(1.02 - 2.70) *	
		Thursday	1.10	(0.65 - 1.88)	
		Friday	1.28	(0.77 - 2.13)	
		Saturday	2.57	(1.62 - 4.08) ***	
		Sunday	3.96	(2.56 - 6.11) ***	
High daily distinct CPI frequency among hospital staff (mean plus three threshold)	Category (ref :HCW)	AHS	0.48	(0.15 - 1.58)	6,00E-12
		Hospital porter	21.14	(9.41 - 47.49) ***	
		Logistic	1.55	(0.33 - 7.19)	
		Physician	5.22	(1.44 - 18.89) *	
		Reeducation staff	1.70	(0.74 - 3.91)	
	days of week (ref: Wednesday)	Monday	1.44	(0.51 - 4.03)	4.80E-14
		Tuesday	1.39	(0.47 - 4.08)	
		Thursday	3.48	(1.35 - 8.95) **	
		Friday	7.88	(3.29 - 18.90) ***	
		Saturday	0.38	(0.04 - 3.21)	
		Sunday	0.00	(0.00 - Inf)	
High daily cumulative duration of CPIs among hospital staff (mean plus three threshold)	Category (ref :HCW)	AHS	1.06	(0.55 - 2.03)	0.002
		Hospital porter	0.00	(0.00 - Inf)	
		Logistic	1.94	(0.72 - 5.17)	
		Physician	0.00	(0.00 - Inf)	
		Reeducation staff	0.10	(0.01 - 0.75) *	
	days of week (ref: Wednesday)	Monday	1.96	(0.88 - 4.37)	0.00037
		Tuesday	1.42	(0.58 - 3.46)	
		Thursday	0.94	(0.35 - 2.56)	
		Friday	2.27	(1.02 - 5.03) *	
		Saturday	2.49	(1.05 - 5.91) *	
		Sunday	4.48	(2.06 - 9.70) ***	
High daily distinct CPI frequency among patients (mean plus three threshold)	Reasons for hospitali	Geriatric	0.85	(0.19 - 3.79)	0.0031
		Neurology	2.05	(0.95 - 4.41)	
		Nutrition	1.35	(0.60 - 3.04)	

	zation (ref: Orthopaedic)	Post-operative	5.01	(2.17 - 11.58) ***	
	Age (ref: [50,60))	[18,30)	1.95	(0.69 - 5.50)	0.88
		[30,40)	1.02	(0.50 - 2.07)	
		[40,50)	1.00	(0.56 - 1.79)	
		[60,70)	1.08	(0.57 - 2.02)	
		[70+]	1.24	(0.61 - 2.50)	
	Gender (ref: Female)	Male	0.98	(0.64 - 1.50)	0.93
	days of week (ref: Wednesday)	Monday	1.26	(0.43 - 3.65)	2.50E-36
		Tuesday	0.49	(0.12 - 1.99)	
		Thursday	5.36	(2.22 - 12.96) ***	
		Friday	10.52	(4.51 - 24.54) ***	
		Saturday	0.00	(0.00 - Inf)	
		Sunday	0.00	(0.00 - Inf)	
High daily cumulative duration of CPIs among patients (mean plus three threshold)	Reasons for hospitalization (ref: Orthopaedic)	Geriatric	7.12	(0.48 - 106.35)	0.014
		Neurology	0.34	(0.09 - 1.15)	
		Nutrition	0.00	(0.00 - 5.53e+55)	
		Post-operative	0.62	(0.13 – 2.96)	
	Age (ref: [50,60))	[18,30)	0.85	(0.11 - 6.43)	2.40E-06
		[30,40)	3.19	(1.74 - 5.84) ***	
		[40,50)	0.72	(0.31 - 1.63)	
		[60,70)	1.79	(0.98 - 3.26)	
		[70+]	0.31	(0.11 - 0.82) *	
	Gender (ref: Female)	Male	1.50	(0.99 - 2.28)	0.058
	days of week (ref: Wednesday)	Monday	2.13	(0.79 - 5.68)	2,00E-06
		Tuesday	1.99	(0.73 - 5.39)	
		Thursday	1.78	(0.65 - 4.91)	
		Friday	2.32	(0.88 - 6.12)	
		Saturday	4.72	(1.92 - 11.60) ***	
		Sunday	6.46	(2.69 - 15.46) ***	

Note: Observed level of the Wald test for each parameter: * <0.05, ** <0.01, *** <0.001. OR: odds ratio.

Supplementary Table S4: Factors associated with high daily contact frequency and high daily cumulative duration with HCW, Physician, Reeducation staff or Hospital porter among patients, resulting from a mixed model with ward-specific random intercepts to account for within-ward and between-ward variations.

	Factor	*level*	*OR*	*CI 95%*	*p-value*
High daily distinct CPI frequency with HCW	Reasons for hospitalization (ref: Orthopaedic)	Geriatric	3.03	(0.77 - 11.87)	1.50E-11
		Neurology	2.37	(1.64 - 3.44) ***	
		Nutrition	0.74	(0.50 - 1.08)	
		Post-operative	4.18	(2.75 - 6.36) ***	
	Age (ref: [50,60))	[18,30)	0.96	(0.54 - 1.71)	4.20E-10
		[30,40)	0.75	(0.58 - 0.98) *	
		[40,50)	1.25	(1.02 - 1.53) *	
		[60,70)	0.99	(0.79 - 1.24)	
		[70+]	2.00	(1.58 - 2.53) ***	
	Gender (ref: Female)	Male	0.84	(0.73 - 0.97) *	0.019
	days of week (ref: Wednesday)	Monday	1.05	(0.85 - 1.30)	1.90E-77
		Tuesday	0.50	(0.40 - 0.64) ***	
		Thursday	0.62	(0.49 - 0.78) ***	
		Friday	0.94	(0.76 - 1.16)	
		Saturday	0.16	(0.11 - 0.22) ***	
		Sunday	0.16	(0.12 - 0.22) ***	
High daily cumulative duration of CPIs with HCW	Reasons for hospitalization (ref: Orthopaedic)	Geriatric	14.42	(3.33 - 62.48) ***	2,00E-12
		Neurology	3.42	(1.83 - 6.40) ***	
		Nutrition	5.64	(2.55 - 12.45) ***	
		Post-operative	6.46	(3.43 - 12.16) ***	

	Age (ref: [50,60))	[18,30)	0.08	(0.01 - 0.60) *	1.30E-22
		[30,40)	0.02	(0.002 - 0.11) ***	
		[40,50)	1.12	(0.83 - 1.52)	
		[60,70)	0.53	(0.36 - 0.78) **	
		[70+]	0.53	(0.34 - 0.80) **	
	Gender (ref: Female)	Male	0.78	(0.62 - 0.97) *	0.029
	days of week (ref: Wednesday)	Monday	1.27	(0.89 - 1.84)	0.029
		Tuesday	1.19	(0.82 - 1.74)	
		Thursday	0.96	(0.64 - 1.42)	
		Friday	0.98	(0.66 - 1.45)	
		Saturday	0.94	(0.63 - 1.41)	
		Sunday	1.61	(1.13 - 2.30) **	
High daily distinct CPI frequency with physician	Reasons for hospitalization (ref: Orthopaedic)	Geriatric	2.05	(0.75 - 5.62)	0.045
		Neurology	1.18	(0.89 - 1.56)	
		Nutrition	0.63	(0.43 - 0.91) *	
		Post-operative	1.12	(0.80 - 1.55)	
	Age (ref: [50,60))	[18,30)	1.26	(0.81 - 1.97)	0.00068
		[30,40)	0.97	(0.72 - 1.30)	
		[40,50)	1.14	(0.88 - 1.48)	
		[60,70)	1.27	(0.97 - 1.65)	
		[70+]	1.82	(1.39 - 2.39) ***	
	Gender (ref: Female)	Male	0.73	(0.62 - 0.86) ***	0.00012
	days of week (ref: Wednesday)	Monday	1.91	(1.47 - 2.47) ***	2.90E-71
		Tuesday	1.28	(0.97 - 1.68)	
		Thursday	2.12	(1.63 - 2.74) ***	

		Friday	1.92	(1.49 - 2.49) ***	
		Saturday	0.31	(0.21 - 0.46) ***	
		Sunday	0.20	(0.13 - 0.31) ***	
High daily cumulative duration of CPIs with physician	Reasons for hospitalization (ref: Orthopaedic)	Geriatric	0.60	(0.07 - 4.74)	0.42
		Neurology	1.78	(0.75 - 4.20)	
		Nutrition	0.78	(0.24 - 2.53)	
		Post-operative	1.78	(0.79 - 3.98)	
	Age (ref: [50,60))	[18,30)	0.51	(0.06 - 3.93)	2.60E-05
		[30,40)	0.31	(0.07 - 1.34)	
		[40,50)	2.05	(1.04 - 4.03) *	
		[60,70)	0.95	(0.36 - 2.47)	
		[70+]	4.48	(2.16 - 9.27) ***	
	Gender (ref: Female)	Male	1.36	(0.79 - 2.34)	0.28
	days of week (ref: Wednesday)	Monday	0.78	(0.28 - 2.14)	2.40E-05
		Tuesday	1.09	(0.42 - 2.82)	
		Thursday	1.74	(0.73 - 4.21)	
		Friday	3.12	(1.41 - 6.89) **	
		Saturday	0.61	(0.20 - 1.84)	
		Sunday	0.33	(0.09 - 1.24)	
High daily distinct CPI frequency with reeducation staff	Reasons for hospitalization (ref: Orthopaedic)	Geriatric	0.49	(0.35 - 0.67) ***	2.80E-06
		Neurology	0.72	(0.59 – 0.89) **	
		Nutrition	1.19	(0.93 - 1.53)	
		Post-operative	0.37	(0.23 – 0.59) ***	
	Age (ref: [50,60))	[18,30)	1.42	(0.92 - 2.19)	0.0052
		[30,40)	1.22	(0.95 - 1.55)	

		[40,50)	0.77	(0.60 - 0.98) *	
		[60,70)	1.05	(0.83 - 1.31)	
		[70+]	0.81	(0.62 - 1.06)	
	Gender (ref: Female)	Male	0.82	(0.71 – 0.96) *	0.015
	days of week (ref: Wednesday)	Monday	1.20	(0.94 - 1.52)	1.40E-140
		Tuesday	1.19	(0.93 - 1.52)	
		Thursday	1.37	(1.07 - 1.74) *	
		Friday	1.75	(1.39 - 2.20) ***	
		Saturday	0.00	(0.00 - 4.63e+67)	
		Sunday	0.00	(0.00 - 2.79e+59)	
High daily cumulative duration of CPIs with reeducation staff	Reasons for hospitalization (ref: Orthopaedic)	Geriatric	1.35	(0.83 - 2.19)	7,00E-04
		Neurology	0.72	(0.51 - 1.02)	
		Nutrition	1.64	(1.09 - 2.45) *	
		Post-operative	0.82	(0.45 - 1.50)	
	Age (ref: [50,60))	[18,30)	0.38	(0.14 - 1.05)	0.01
		[30,40)	0.58	(0.37 - 0.90) *	
		[40,50)	0.58	(0.40 - 0.86) **	
		[60,70)	0.61	(0.42 - 0.89) *	
		[70+]	0.65	(0.42 - 1.01)	
	Gender (ref: Female)	Male	1.10	(0.87 - 1.39)	0.44
	days of week (ref: Wednesday)	Monday	1.63	(1.12 - 2.38) *	1.80E-47
		Tuesday	1.43	(0.96 - 2.11)	
		Thursday	1.47	(1.00 - 2.17)	
		Friday	1.83	(1.26 - 2.66) **	

		Sunday	0.00	(0.00 - 7.57e+146)	
		Saturday	0.00	(0.00 - 4.31e+88)	
High daily distinct CPI frequency with hospital porter	Reasons for hospitalization (ref: Orthopaedic)	Geriatric	0.33	(0.16 - 0.68) **	3.10E-10
		Neurology	0.70	(0.54 - 0.92) **	
		Nutrition	0.58	(0.43 - 0.78) ***	
		Post-operative	0.37	(0.26 - 0.52) ***	
	Age (ref: [50,60))	[18,30)	0.94	(0.62 - 1.40)	0.71
		[30,40)	1.18	(0.95 - 1.46)	
		[40,50)	1.07	(0.88 - 1.29)	
		[60,70)	1.01	(0.83 - 1.23)	
		[70+]	1.03	(0.82 - 1.29)	
	Gender (ref: Female)	Male	0.78	(0.69 - 0.89) ***	0.00026
	days of week (ref: Wednesday)	Monday	1.09	(0.90 - 1.32)	1.60E-265
		Tuesday	1.06	(0.87 - 1.29)	
		Thursday	1.11	(0.91 - 1.35)	
		Friday	1.28	(1.05 - 1.55) *	
		Saturday	0.00	(0.00 - 4.11e+39)	
		Sunday	0.00	(0.00 - 0.94) *	
High daily cumulative duration of CPIs with hospital porter	Reasons for hospitalization (ref: Orthopaedic)	Geriatric	0.30	(0.18 - 0.49) ***	0.009
		Neurology	0.82	(0.59 - 1.13)	
		Nutrition	1.13	(0.76 - 1.66)	
		Post-operative	0.79	(0.43 - 1.46)	
	Age (ref: [50,60))	[18,30)	0.63	(0.25 - 1.60)	0.008

	[30,40)	1.12	(0.73 - 1.70)	
	[40,50)	1.62	(1.15 - 2.30) **	
	[60,70)	1.09	(0.74 - 1.59)	
	[70+]	1.70	(1.16 - 2.50) **	
Gender (ref: Female)	Male	0.62	(0.48 - 0.79) ***	0.00013
days of week (ref: Wednesday)	Monday	0.92	(0.62 - 1.38)	9.20E-49
	Tuesday	1.11	(0.75 - 1.64)	
	Thursday	1.27	(0.87 - 1.85)	
	Friday	1.9	(1.34 - 2.70) ***	
	Saturday	0.00	(0.00 - Inf)	
	Sunday	0.00	(0.00 - Inf)	

Note: Observed level of the Wald test for each parameter: * <0.05, ** <0.01, *** <0.001. OR: odds ratio.

Supplementary Figure S1: Time trends in CPIs over a 24-hour day for each day of the week during the whole study period. Boxplot of the distribution of hourly CPI

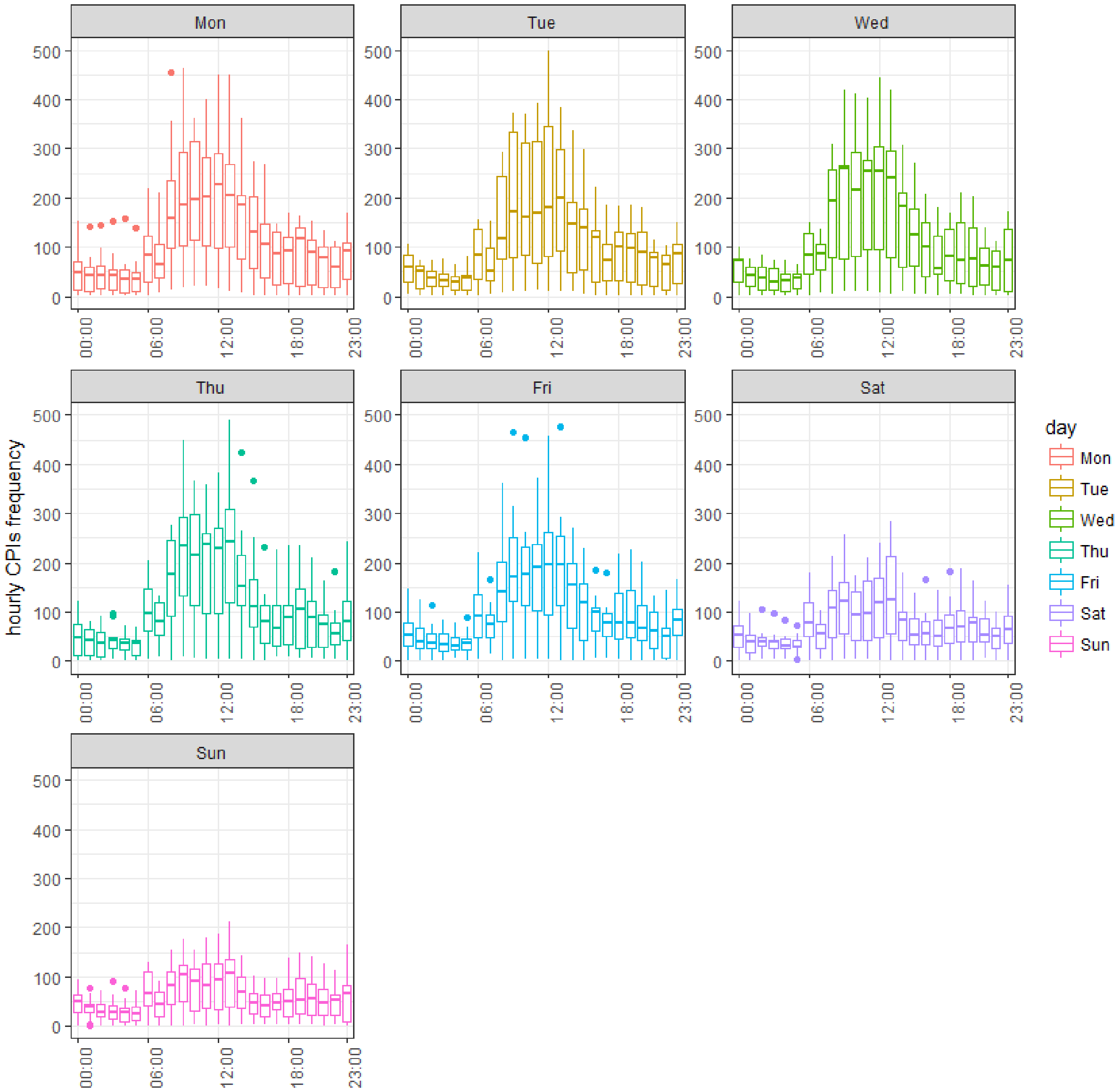
Mon
Tue
Wed
Thu
Fri
Sat
Sun
hourly CPIs frequency
0
100
200
300
400
500
00:00
06:00
12:00
18:00
23:00
day
Mon
Tue
Wed
Thu
Fri
Sat
Sun
